# Exploring young Australians’ understanding of sustainable and healthy diets: a qualitative study

**DOI:** 10.1017/S1368980022001513

**Published:** 2022-10

**Authors:** Rimante Ronto, Golsa Saberi, Julia Carins, Keren Papier, Elizabeth Fox

**Affiliations:** 1 Department of Health Systems and Populations, Faculty of Medicine, Health and Human Sciences, Macquarie University, NSW 2109, Australia; 2 Social Marketing @ Griffith, Griffith University, QLD 4111, Australia; 3 Epidemiology Unit, Nuffield Department of Population Health, University of Oxford, Oxford, UK; 4 Department of Population Medicine and Diagnostic Sciences, Cornell University, Ithaca, USA

**Keywords:** Sustainable and healthy diets, Young adults, Food choice, Qualitative research

## Abstract

**Objective:**

This qualitative study aimed to explore young Australians’ perspectives, motivators and current practices in achieving a sustainable and healthy diet.

**Design:**

Semi-structured online interviews were conducted with young Australians. Interviews were audio-recorded using the online Zoom platform, transcribed and analysed using a deductive analysis method by applying the Theoretical Domains Framework and inductive thematic data analysis.

**Setting:**

Young Australians recruited via social media platforms, noticeboard announcements and flyers.

**Subjects:**

Twenty-two Australians aged 18 to 25 years.

**Results:**

The majority of participants were aware of some aspects of a sustainable and healthy diet and indicated the need to reduce meat intake, increase intake of plant-based foods, reduce food wastage and packaging and reduce food miles. Young adults were motivated to adopt more sustainable dietary practices but reported that individual and environmental factors such as low food literacy, limited food preparation and cooking skills, lack of availability and accessibility of environmentally friendly food options and costs associated with sustainable and healthy diets hindered their ability to do so.

**Conclusions:**

Given the barriers faced by many of our participants, there is a need for interventions aimed at improving food literacy and food preparation and cooking skills as well as those that create food environments that make it easy to select sustainable and healthy diets. Future research is needed for longitudinal larger scale quantitative studies to confirm our qualitative findings. In addition, the development and evaluation of individual and micro-environmental-based interventions promote sustainable and healthy diets more comprehensively.

Our current global food systems are not environmentally sustainable, with about 21–37 % of the total greenhouse gas emissions attributed to the food system from agriculture and land use change, food production, retail and consumption^(1–3)^. Many high-income countries disproportionately contribute to these environmental outcomes^(4)^. At the same time, globally, dietary intake is not aligned with recommended dietary practices. On a global scale, 11 million deaths and 255 million disability adjusted life years were related to dietary risk factors (e.g. low intake of whole grains, fruits and vegetables and high consumption of salt) in 2017^(5)^. In Australia, 20 % of all premature deaths from non-communicable diseases were attributed to poor diet^(6)^. Dietary behaviours are both the result and driver of our food systems^(7)^, and current diets are leading contributors to the global burden of non-communicable diseases, including premature mortality from CVD, diabetes and certain type of cancers^(4)^.

In order to achieve positive outcomes for human and environmental health, diets that are both healthy and environmentally sustainable are needed. The FAO of the United Nations defines sustainable diets as *‘those diets with low environmental impacts which contribute to food and nutrition security and to healthy life for present and future generations. Sustainable diets are protective and respectful of biodiversity and ecosystems, culturally acceptable, accessible, economically fair and affordable; nutritionally adequate, safe and healthy; while optimising natural and human resources’*^(3)^. The EAT-Lancet Commission was tasked to define the components of a sustainable and healthy diet; this ‘planetary health diet’ consists largely of plant-based foods (e.g. wholegrains, vegetables), low amounts of animal sourced foods (red and processed meat in particular) and little to no added sugars, refined grains and ultra-processed foods^(4)^. Animal-derived food production has the highest negative impact on environment^(8)^ and health as a recent systematic literature review and meta-analysis found that higher consumption of unprocessed red meat was associated with a 9 % higher risk, and processed meat intake with an 18 % higher risk of Ischaemic Heart Disease^(9)^.

Although growing evidence suggests that a healthy diet is environmentally sustainable and is associated with better health outcomes^(4,10)^, and in spite of dietary guidelines existing to promote healthy diets, most Australians fail to meet Australian Dietary Guidelines for Healthy Eating^(11)^. The 2017–2018 Australian National Health Survey showed that only 5 % of Australian adults met fruit and vegetable recommendations^(12)^. Furthermore, Australian adults exceeded total energy intake requirements and the recommendations for weekly red meat intake in 2011–2012^(13)^. Finally, over one-third (35 %) of total energy consumed in Australia was from discretionary foods which are considered low in nutritional value and high in saturated fats, sugar and salt^(13)^.

In order to develop effective policies and interventions to change our unhealthy and unsustainable dietary behaviours, there is a need to explore consumers’ behaviours and motivation for achieving more sustainable dietary practices. A few studies have explored Australians’ knowledge, awareness and attitudes towards healthy and environmentally friendly dietary patterns^(14–17)^. These found that Australians have limited understanding of the impact of their dietary behaviours on the environment with some believing their diets had a trivial effect on the environment^(14–17)^. However, one study indicated that youth may have more environmentally friendly intentions, such as considering consumption of less meat and more organic local products, being more open to increasing vegetarian dietary patterns and recycling food waste^(17)^. Young adults can be powerful drivers in their families and communities^(18–20)^ in terms of driving the demand for healthier and/or environmentally friendly food consumption.

Therefore, the current study aimed to explore young Australians’ perspectives, motivators and current practices in achieving a sustainable and healthy diet. The findings from this research will be used to guide possible interventions that could help young Australians (as well as other Australians in their communities) achieve healthier and more environmentally sustainable diets.

## Methods

### Participants

Young Australians formed the study sample. Young or emerging adults refer to individuals aged 18 to 25 years^(21)^. Participants were recruited using convenience sampling through social media platforms (e.g. Facebook, Instagram, Twitter, etc.), noticeboard announcements through (removed for double-blind reviewing) University Newsletters, councils’ notice boards and flyer distribution across (removed for double-blind reviewing) University and gyms. Recruitment of the sample was conducted in August–September 2020. An informed consent form was provided to all participants and if they agreed to take part in the study, an interview was scheduled at a time and day most suitable to the participant. (Removed for double-blind reviewing) University, Human Research Ethics Committee granted the approval for the current study (Reference No: 52020793218854). Participants were offered a $20 Woolworths Gift Card for their participation.

### Study design and data collection

A qualitative study was undertaken using semi-structured interviews as the data collection method to explore young Australians’ views on sustainable and healthy diets, current practices, and enablers and barriers associated with achieving sustainable and healthy diets. All semi-structured interviews were conducted through a digital platform (Zoom). Participants were asked to send three pictures of their dinners prepared on different days (either weekdays or weekend) to facilitate discussion during the interview. All interviews were conducted by one interviewer and lasted between 30 and 45 min.

A series of open-ended interview questions with additional probing questions were developed by the research team (see Supplementary file 1). Questions were grouped in the following themes: (i) understanding of a sustainable and healthy diet concept; (ii) the role of different foods on health and the environment and (iii) barriers and enablers of achieving sustainable and healthy diets. In addition, some basic socio-demographic questions (e.g. age, sex, education and employment status) were asked.

### Data analysis

All digital recordings were professionally transcribed using Rev.com services, and one researcher reviewed the transcripts for accuracy against the original audio recordings. Data analysis was conducted concurrently with data collection to allow both to mutually shape each other and decide on theoretical data saturation. The theoretical saturation was reached at interview 20, where no new data or constructs of interest emerged^(22)^.

Thematic data analysis was used to analyse the data. Both deductive and inductive reasonings were used to analyse the data. Deductive reasoning was used to categorise the data into the Theoretical Domains Framework constructs. However, not all data from the interviews fit within the TDF. Therefore, inductive reasoning was applied to form the subthemes and overall themes. First, the TDF was used to deductively develop an initial coding framework^(23)^. The TDF incorporates constructs from a range of behaviour change theories and has been used to identify potential target areas for the development of successful interventions by identifying barriers and enablers^(24,25)^. It includes fourteen constructs: Knowledge, Skills, Social/Professional Role and Identity, Beliefs about Capabilities, Optimism, Beliefs about Consequences, Reinforcement, Intentions, Goals, Memory, Attention and Decision Processes, Environmental Context and Resources, Social Influences, Emotions and Behavioural Regulation. Two researchers manually coded the data and categorised the data into the TDF constructs (see Supplementary file 2). After the data were coded, the researchers inductively collated the coded information into subthemes and themes. All researchers reviewed these themes in order to increase the trustworthiness of the findings^(26)^.

## Results

In total, twenty-two young Australians (aged between 21 and 25 years) participated in the current study, of which two-thirds were women (see Table 1). The majority of participants (82 %) were single, employed either on a part-time or casual basis (68 %) and were highly educated (50 % with a high school degree and 50 % current undergraduate students). Furthermore, 65 % of participants reported not following any dietary restriction, while 30 % of participants indicated that they followed either a vegetarian, vegan or pescatarian dietary pattern; one participant indicated having food allergy which impacted their dietary pattern. Approximately 18 % of participants experienced food insecurity in the last few weeks, mainly due to the COVID-19 pandemic and associated loss in income. Finally, two-thirds of participants (67 %) were aware of some aspects of a sustainable and healthy diet concept, but only 45 % of participants tried to practise it in some way (e.g. reduced red meat intake).

**Table 1.** Socio-demographic characteristics of the sample (*n* 22)

Socio-Demographic characteristics		*n*	%
Sex	Men	5	23
	Women	17	77
Age	18–20 years	6	28
	21–23 years	8	36
	24–25 years	8	36
Marital status	Single	18	82
	Married	1	5
	In relationship	3	13
Employment status	Unemployed	5	23
	Casual	12	54
	Part-time	3	14
	Full-time	2	9
Education	High school	11	50
	Undergraduate	10	45
	Postgraduate	1	5
No. of household people/children	≤ 4 people	20	91
	≥ 5 people	2	9
Dietary restrictions	None	13	65
	Vegetarian/vegan diet	4	20
	Pescatarian	2	10
	Allergies	1	5
Experienced food insecurity in the last few weeks	No	18	82
	Yes	4	18
Awareness and practicing of sustainable and healthy diets	Aware and practicing	10	45
	Aware but do not practice	5	23
	Not aware/very limited awareness	7	33

### Themes

The participants’ data on their understanding of sustainable and healthy diets and current efforts in achieving it were categorised mainly within eight TDF constructs: *‘knowledge’*, *‘behavioural regulation’*, *‘environmental influences’*, *‘social influences’*, *‘emotion’*, *‘beliefs about consequences’*, *‘social professional role and identity’* and *‘skills’* (see Supplementary file). Four major themes were formed from the categorised data: (i) Understanding of sustainable and healthy diets; (ii) skills and motivation in achieving a sustainable and healthy diet; (iii) actions taken towards achieving a sustainable and healthy diet and (iv) enablers and barriers in achieving a sustainable and healthy diet. The themes and sub-themes are described in detail below and illustrative quotes presented in Table 2.

**Table 2.** Themes, subthemes and illustrative quotes

Theme	Subtheme	Illustrative quotes
Understanding of sustainable and healthy diets	Foods for health and environmental sustainability	*‘Healthy is more about making sure that all your food has the right nutrients, and that you’re not eating too much foods that have really high amounts of salt or sugar, or unhealthy fats*’*(P6).* *‘Sustainability in your diet, to me, is about where does your food come from? And how many miles has it come to travel to you? But then also, whether it’s really sustainable to grow something in that climate*’*(P6).* *‘Healthy is in meeting all the needs of your body. It’s something that is not only physical health, but also mental health. You could survive off porridge and potatoes for the rest of your life, but at the end of the day, you’re not going to be happy doing that. So I think a very diet plays into the physical and mentally healthy role. As for sustainable, I sort of see that as cutting back on meat and sourcing things from local producers*’ *(P1).* *‘So healthy usually it means not buying processed food. Making as much as you can by yourself with as many fresh ingredients as possible. So if I were to eat healthy I would mostly buy fresh fruits and vegetables, make a curry out of that, make my own lentils, make rice by myself. Making it by myself, not processed, basically*’*(P15).* *‘If you want the diet to be sustainable, in my opinion, it has to be something which is true to your roots, I mean, from whichever ethnic background you’re from. If that’s what you’re used to eating from your childhood, you cannot just completely change. If you have been a non-vegetarian your whole life, you cannot turn into a vegan overnight*’ *(P4).*
	Sustainable diet on the plate	*‘I would usually have one cup of rice or carbs, and then I would have protein as well, so that would be either fish or chicken or eggs if I wanted to. And then the most of it would be veggies. Yeah, veggies. So that’s how I would plate out my dinner*’ *(P16).*
	Awareness of the relationship between diet, health and the environment	*‘Not really* (heard of sustainable diet)*. If I had, I didn’t take it into notice or maybe I had it in passing and I didn’t really value the discussion*’ *(P14).* *‘A movie that I saw last week, about how avocados were taking up a huge amount of the water in Chile, and a lot of people didn’t have access to water because of the way that it was being produced for wealthier countries. And then, I know that a lot of food production is made in a way that’s not sustainable, is dangerous to the people who live in those areas*’*(P6).* *‘I tend to avoid animal-based products, and I guess food crops that require extensive land, water, or land or water use. Particularly almonds, coconuts, quinoa, a few more. But like those kind of food groups I tend to avoid as much as I can*’*(P7).* *‘I am actively trying to cook with less meat and buy less meat because I know that industry produces a lot of greenhouse gases*’*(P1).* *‘I do know that meat is not very sustainable, especially beef, it takes up a lot of resources. So it would definitely be on the vegetarian side, and I guess equal proportions of carbs, vegetables, and you would need protein as well*’*(P15).* *‘I tend to avoid animal-based products, and I guess food crops that require extensive land, water, or land or water use. Particularly almonds, coconuts, quinoa, a few more. But like those kind of food groups I tend to avoid as much as I can*’*(P7).*
Skills and motivation in achieving sustainable and healthy diet	Food preparation and cooking skills	*‘I’ve been cooking for myself for most of my life, because, well, I’m vegetarian.*’ *(P6).* *‘When I cook, usually I cook for two days at one time, especially if I’m really busy. So if I cook for two days at one time, it helps me not to eat unhealthy like noodles and stuff. That really helps, I guess*’*(P15).*
	Motivated to eat healthy	*‘Knowing that in the future my health is going to be pretty dependent on how I choose to live my life now. I know that the older you get, the more difficult it is to maintain a healthy lifestyle if you haven’t started younger*’*(P6).* *‘When I’m eating healthy, I think of, oh, it’s for my long term health because I don’t want to be sick when I’m old and that’s about it*’*(P9).* *‘I try avoiding beef, even though I eat beef all the time, but I do eat beef a little less because I know that beef cultivation is still harmful towards our environment and I’m very against animal abuse*’*(P10).*
	Feeling good when eating for health and environment	*‘I feel really good when I don’t eat a lot of meat. I felt really not healthy, but very like light and sluggish and not so drained*’*(P3).* *‘I feel really bad mentally if I eat like chicken or pork or beef, maybe like two to three consecutive days. It happens only if I’m out. So, if I’m with my family living, we have the home cooked meal, which is always vegetarian. But, if I’m visiting uni and I’m meeting people, so I might consume something which has non-veg in it, so I would feel really bad. And maybe psychologically for the environment and also for my body that I’ve consumed a lot of meat. So, what I would usually do that I won’t eat meat for next month*’*(P8).* *‘Just the fact that I’m very passionate about sustainability and the environment. It’s one of the things that I care the most about. I guess it’s just empathy for nature, and, yeah, knowing that I have an impact and I can do something, I feel like I should. I have that sense of responsibility*’*(P13).* *‘I don’t want to be in a position where I feel guilty about foods that I’m eating, or be overly conscious about things like that*’*(P6).* *‘So, I do feel better. It’s part of the mind as well, you feel guilty when you eat junk food, but you do notice it in your energy levels. I don’t feel as much bloated*’ *(15).* *‘If people are told more about healthy diets, they would really consider taking it. But there’s also a limitation in what people eat, depends on what schedule they have. So people can really embark on a healthy diet when they don’t have the time to prepare*’*(P14).*
Actions taken towards achieving sustainable and healthy diet	Reducing intake of less healthy foods	*‘I am trying to eat foods that don’t have a lot of sugar content, a lot of bad fats in them. So for example, maybe you choose olive oil over butter or something like that. Have avocados instead*’*(P2).* *‘My weight. I know that the BMI range that I’m in, it’s not in the normal side, it’s slightly overweight, so I am trying to cut down my food or trying to incorporate more healthier choices*’*(P4).*
	Reducing animal-derived and resource intensive foods	*‘So, I don’t eat much dairy. I don’t eat much red meat, and I try to eat a lot of fish. And I don’t often have egg by itself, but sometimes egg is included in preparation for my foods*’*(P17).* *‘I heard that actually planting avocados will harm the environment. I’m not exactly sure, but I mean, it’s another reason for me to reduce eating avocados because it seems avocados are quite pricey and it’s another reason to reconfirm that, oh, I should reduce the intake of avocados and try to replace it with other sources and then more sustainable sources*’*(P9).*
Enablers and barriers in achieving sustainable and healthy diet	Healthy and environmentally sustainable food costs more	*‘There’s a lot of factors so based on what’s on special, on discount on the market. And also based on what’s available in my fridge and also based on how much time do I have to prepare the meals*’*(P9).* *‘As a student on a student budget, I had to cut down on fish, so that’s when the major change happened*’ *(P4).* *‘Last few months with COVID, yeah, that I lost my job and everything. I got help from the uni. I got food bank and all of that*’*(P13).* *‘I’m pretty sure when I moved back to Sydney, when this COVID thing settles, and I have a proper job in Sydney, I have made a promise to myself that I’ll cook for myself. It doesn’t matter if I’m living alone, and I will eat properly just like I eat properly when I’m here with my family*’*(P8).*
	(Un)Availability of healthy and environmentally sustainable food	*‘When I go out to eat in restaurants, most restaurants in Sydney aren’t very vegetarian friendly, so they’ll have some vegan friendly menus that have some substitutes for meat*’*(P15).* *‘I’m trying to eat less sugar. It’s not necessarily working, but I am aiming to eat less sugar, and sometimes my mom will sneak a few vegetables into my plate, something like sliced carrots and stuff, and so I try and make an effort to eat more of those, get second helpings of those as well. But I don’t think the healthiness of foods really regulates what I eat as much as it should.*’ *(P18).* *‘What’s available in the fridge because I don’t really like to go out to eat. So if there’s anything at home that I can eat, I’ll just eat it. So I guess availability of food at home*’*(P16).* *‘Also it depends on where I buy my foods. Let’s say if for whatever reason, I need to travel out to the countryside or out to the central coast area, where there might not be a major supermarket or that provides, or a general local farmers’ market that provides affordable food and vegetables and fruits and grains and such*’ *(P7).* *‘So sometimes when you’re out with your family or friends and having a lot of unhealthy foods because I’m a human, So definitely I feel like eating them*’ *(P9).* *‘When I go out and I’m socialising with friends, it’s a bit hard to pick the healthy option*’ *(P16).*
	Positive and negative role modelling	*‘And because my friends are vegetarian vegan as well, so I don’t eat red meat around them*’ *(P17).* *‘I am trying to eat a lot less meat. I know that it’s hard because I grew up in a very meat heavy family (P1).* *‘Since my parents don’t allow meat to be cooked in the home, in the kitchen, we do use a soy mince replacement, if we’re trying to make something with beef mince or anything*’ *(P18).*
	Availability of information sources and its impact on food choices	*‘So I try my best to look up on Google. So I know what information, what websites present. So it’s something presented in Forbes. I would, I would more likely to be believing it, but then something presented like other reps that I’ve never heard of. Or if the website seems very shade, you’re sketchy. I usually don’t mind*’*(P10).* *‘But luckily university helped me understand that it’s just important to just critically think and look at both side of the arguments and decide from there*’*(P7).* *‘It’s a lot of public health campaigns but eat the rainbow, so making sure you have a good variety of predominately 80 % of fresh produce*’*(P3).* *‘First it was the recipes. I’m not too sure how to incorporate vegetables on this much amount into my meals*’*(P11).*

### Theme 1 – Understanding of sustainable and healthy diets

#### Foods for health and environmental sustainability

The majority of participants described a healthy diet as being balanced in terms of including all food groups and appropriate distribution of macronutrients (carbohydrates, proteins and fats). (TDF-*‘Knowledge’*). For instance, one participant stated:

*‘I’d say a diet that’s balanced, and has a good amount of vegetables and fruits included in it, as well as meat, like lean meat and a little water as well, though’ (P19).*

They also emphasised that reduction in salt, sugar, unhealthy fats and discretionary foods are important in achieving a healthy diet. Participants stated that diets aligned with environmental sustainability involved moderation and appropriate portion sizes (i.e. not overconsuming foods), increasing vegetable consumption, reducing consumption of processed foods and meat and reducing food waste. Some participants emphasised that foods should be organic and grown without pesticides or fertilisers. In addition, participants mentioned that it is important that foods are ‘*fresh*’ and preferably locally produced in order to reduce food miles. Finally, a few participants stated that they tried to reduce consumption of certain foods that require significant resources to grow or produce, for example nuts (almonds, coconuts and avocados) in order to reduce environmental footprint. (TDF-*‘Knowledge’*). As one participant explained,

*‘Sustainable diet would mean not just what you’re eating, but also where it’s produced, how much packaging it has. And I guess healthy is sort of along the same lines as that, so if it’s organic, the fat content, sugar content, salt content, things like that. And also how much of it you’re eating. So I think moderation is a big part of sustainable and healthy. Eating healthily would mean to try and consume a lot of vegetables, drinking a lot of water, eating fruit but not too much of it, staying away from processed foods or foods high in sugar, salt, fat, and also trying to have a balanced diet of your proteins and things like that’ (P5)*

A healthy and sustainable diet also involved more than just physical health and environmental sustainability. A few participants stated that it is important that their diet or the foods they consumed made them feel good and were *‘true to your (their) roots’* (referring to cultural background. (TDF-*‘Social professional role and identity’*).

*‘If you want the diet to be sustainable, in my opinion, it has to be something which is true to your roots, I mean, from whichever ethnic background you’re from. If that’s what you’re used to eating from your childhood, you cannot just completely change. If you have been a non-vegetarian your whole life, you cannot turn into a vegan overnight’ (P4).*

### Sustainable diet on the plate

Most participants explained the proportions of a sustainable and healthy diet on the plate as: half of the plate should be vegetables or ‘greens’, one quarter of ‘carbs’ (referring to grains such as rice, pasta, bread) and one quarter of protein (referring to meat- and plant-based proteins such as beans). (TDF-*‘Knowledge’*).

*‘Would be, think about half the plate would be vegetables and then one quarter of it would be pasta or rice carbohydrates and one quarter will be meat, I’m cooking’ (P11).*

Participants who mentioned meat stated that only a small portion of red meat was needed for a healthy diet or that there was a need to switch to more sustainable sources of protein, for example fish, chicken, eggs or other plant-based proteins such as beans. (TDF-*‘Knowledge’*). For instance,

*‘I’m not too knowledgeable, but I do know that meat is not very sustainable, especially beef, it takes up a lot of resources. So it would definitely be on the vegetarian side, and I guess equal proportions of carbs, vegetables, and you would need protein as well. So something more sustainable than meat would be protein or lentils’ (P15).*

One participant also mentioned that this may not be applicable when going out to eat where health and environmental considerations may be compromised. (TDF-*‘Environmental influences’*). As one participant noted:

*‘I’d definitely try for a quarter to a half of vegetables, a quarter of protein, and then a quarter of carbohydrates. Definitely what I aim for. We definitely try to have at least two to three servings of veggies per dinner, except for, of course, when we go out’ (P12).*

### Awareness of the relationship between diet, health and the environment

Ten participants were aware of some aspect of sustainable diets as defined by the EAT-Lancet Commission, and they were actively adhering to such dietary practices by removing or limiting meat in their diet. Five participants were aware of sustainable diets, but they were not practising it, due to either not being ready for a change or the lack of knowledge on how to achieve it. Seven participants were either not aware or had very limited awareness of a sustainable diet and were not practising it. (TDF-*‘Knowledge’*, *‘Behavioural regulation’*).

*‘Impact of different foods (on the environment)? I’ve heard of it, but I’m not exactly… I don’t really understand what’s the mechanism behind them’ (P9).*

Some participants understood that some foods require a lot of resources to be produced. (TDF-*‘Knowledge’*). For instance,

*‘So, I specifically know that beef, like cattle is very heavy on the environment in terms of CO2 emissions, I think it is. And a lot of the growing of crops in raising of cattle requires a lot of water’ (P20).*

These participants specifically identified that some types of meat (beef) and some vegetables (avocados) required intensive resources, including water. (TDF-*‘Knowledge’*). They tried to avoid or limit intake of these foods as much as possible. (TDF-*‘Behavioural regulation’*). Although participants could identify many foods that are often cited as resource-intensive, they also cited some foods as resource-intensive that are not known to require many resources (e.g. quinoa). (TDF-*‘Knowledge’*).

*‘I tend to avoid animal-based products, and I guess food crops that require extensive land, water, or land or water use. Particularly almonds, coconuts, quinoa, a few more. But like those kind of food groups I tend to avoid as much as I can*’*(P7).*

### Theme 2- Skills and motivation in achieving a sustainable and healthy diet

#### Food preparation and cooking skills

Cooking was a distinct skill among half of the participants. Some participants increased their cooking skills mainly when they changed their dietary behaviours. For example, they became a vegetarian, wanted to eat healthier or reduced meat intake, so they needed to cook for themselves to achieve those diets. However, a few participants reported having basic or no cooking skills or not being interested in cooking. (TDF-*‘Skills’*).

*‘I’ve been cooking for myself for most of my life, because, well, I’m vegetarian.’ (P6).*

‘*Because I’m not a very good cook. And when I was living by myself, I didn’t really know what to cook*’*(P17).*

Some of the participants with cooking skills reported having good food preparation and planning skills. They prepared meals in advance, so they could eat healthily if they had some time pressures. Participants reported learning how to cook from their mothers, home economics classes at school or by teaching themselves. (TDF-*‘Skills’*, ‘Goals’, *‘Behavioural regulation’*, *‘Social influences’*).

*‘In my year 11, 12, I have studied home science. And in home science, we used to learn all about food preparation meals, what should we eat, what shouldn’t we eat. And that was the time I really get into like all the food. And that was really interesting subject for me. And we should have cooking practicals as well. And we have learned all about meal preparation, what should we eat, how should we eat, and how should we design our plate and stuff like that. So I have learned my first basic base of knowledge of food and diet and information was from my home science subject*’*(P8).*

Some participants reported that they would like more recipes to be available on how to cook sustainable and healthy meals as they reported this as being a major barrier to consuming sustainable and healthy meals. (TDF-*‘Skills’*).

*‘There’s not a variety of like recipes for me, for example, tofu, I only know couple of recipes. Um, but yeah, I think that’s the biggest barriers for me*’*(P21).*

### Motivated to eat healthy

One-third of participants were motivated to avoid or eat less ultra-processed foods for health reasons and to eat healthily in general to avoid developing non-communicable diseases later in life. They were also motivated to replace processed meat with fresh fruits and vegetables. Some participants were highly motivated to eat less meat for health reasons, stating that eating less meat is healthy for their body, including skin and digestive system. (TDF-*‘Knowledge’*, *‘Beliefs about consequences’*). For instance,

*‘Red meats, I feel like aren’t as good for my health or my digestion than chicken or fish is, so I’ll try and have those more. Tofu is a big component*’*(P2).*

Other participants indicated that they were motivated to eat less meat for environmental reasons or due to concerns about animal welfare. However, one participant explicitly reported not being motivated to reduce meat intake at all, as eating meat was enjoyable. (TDF-*‘Knowledge’, ‘Emotion’*).

### Feeling good when eating for health and environment

Physical and mental health was an important aspect of dietary choices. Some participants reported that they were motivated to eat healthier as it gave them energy and helped them to be active and ‘*fresh*’ during the day. (TDF-*‘Emotion’*). For instance, one participant remarked:

*‘It has effects on your energy levels. So I prefer that I’m like more energetic than tired all the time. I know straight off, right faster to get tired, straight away’ (P19).*

The majority of participants stated that they felt good physically and/or mentally when they ate healthy food. Some participants stated that switching from red meat to white meat or less meat in general made them feel better. (TDF-*‘Emotion’, ‘Behavioural regulation’*).

*‘I feel really good when I don’t eat a lot of meat. I felt really not healthy, but very like light and sluggish and not so drained*’*(P3).*

Moreover, about half of the participants reported that they felt good if their eating decisions contributed to the environment as well. Participants reported feeling bad or guilty if they ate less healthy food. Participants stated being frustrated if they were being criticised on what they ate. (TDF-*‘Emotion’*). As one participant explained,

*‘I guess when I get criticism from other people, whether they’re right or not, or whether they got their sources from somewhere, which I may or may not know. And I guess sometimes being criticised what I eat. That may a bit of a frustration. Because it’s a negative connotation of being a vegan is some sort of green hippie shenanigans. And I guess the social aspect can be a bit rough. And I guess if I was younger than might have deferred me from continuing or thinking about sustainable practice. But luckily university helped me understand that it’s just important to just critically think and look at both side of the arguments and decide from there*’*(P7).*

### Theme 3 – Actions taken towards achieving sustainable and healthy diet

#### Reducing intake of less healthy foods

The majority of participants indicated that they were trying to reduce their consumption of ultra-processed and junk foods. Many focused on reduction of foods containing unhealthy fats, refined carbs (sugar), salt and canned fish, given concerns about their impact on health. (TDF-*‘Goals’, ‘Behavioural regulation’*). As one participant explained,

*‘I’ve been trying to eat less canned fish just because of reports I’ve heard of microplastics and heavy metal in them, the accumulation in them*’*(P1).*

Participants also cited trying to reduce intake of less healthy foods in order to stay within the normal range for BMI.

### Reducing animal-derived and resource-intensive foods

As noted above, participants mentioned that they were actively trying to reduce consumption and purchase of resource-intensive products. Some participants were concerned about packaging and plastics and stated that they were trying to reduce foods that had lots of packaging. (TDF-*‘Behavioural regulation’*).

*‘I’m definitely someone who’s very conscious of packaging and things like that. I tend to not use packaged items if I can. And on the environment as a whole*’*(P20).*

Additionally, about two-thirds of participants indicated that they were trying or had reduced animal-derived foods, such as red meat, dairy and eggs. Some participants stated that they were reducing meat intake in their diet, but they were not able to cut meat out of their diet completely. (TDF-*‘Goals’, ‘Behavioural regulation’*). For instance,

*‘I never cut myself off red meat completely. I’ve reduced my intake of it over time, but it’s not necessarily that I really consumed a lot more. So it never really had a big effect. So let’s suppose I was consuming it four times a week. I cut it down to once or twice a week, but didn’t have it that big of an effect on my personal physique or my mental state, nothing at all*’*(P10).*

Some participants negotiated their intake over time. For instance, one participant stated that if they ate more meat than usual in one week, then they reduced it in the following week. This was their way of ‘*balancing it*’. (TDF-*‘Behavioural regulation’*).

### Theme 4- Enablers and barriers in achieving a sustainable and healthy diet

#### Healthy and environmentally sustainable food costs more

Many participants believed that most people lacked knowledge about sustainable and healthy diets, and if they knew more about it they would switch to healthier and more sustainable food choices. However, one participant was less positive, stating that people do not care about the environment, and all they think about is the ‘*price tag’* and their time availability. (TDF-*‘Knowledge’, ‘Optimism’, ‘Environmental influences*).

*‘No one cares about sustainability, no one really cares about like where the food is coming from. Everybody is looking at the price tags and everybody’s looking at the marketing words, the high words*’*(P10).*

Half of the participants stated that they perceived healthy and sustainable food to cost a lot more than less healthy and/or sustainable food options. They stated that buying or preparing healthy and sustainable food such as plant-based options/alternatives, fresh vegetables or dining at a restaurant (e.g. a vegan restaurant) were more expensive. Some participants stated that they lived on *‘a student budget’*. Therefore, they focused more on the price of the food and often bought discounted options or products on special offer. (TDF-*‘Environmental influences’*).

*‘I would say that it is very hard to maintain a healthy and sustainable diet, because even if you do wish to eat healthier, more sustainable, it is usually more expensive, and as students, it is very hard to maintain that*’*(P15).*

Socio-economic status had an impact on healthy and environmentally sustainable food options available to participants. For example, some participants reported that their families were financially secure so they could afford to buy healthier foods. (TDF-*‘Social and Environmental influences’*).

*‘I know that I come from a very privileged background. So, I know I have the funds to eat healthy to my standard of healthy, which is quite nice. And I have the access to the food that I want. There’s no limitations on where I can get it from or … There’s four supermarkets I can access very easily. I don’t have to worry about that. And I think that I just have minimal barriers to eat healthy*’*(P12).*

Some participants reported losing their jobs due to the COVID-19 pandemic and faced financial constraints. Therefore, at this stage, their priority was price rather than health and sustainability aspects when choosing foods. (TDF-*‘Social and Environmental influences’*).

*‘I’m pretty sure when I moved back to Sydney, when this COVID thing settles, and I have a proper job in Sydney, I have made a promise to myself that I’ll cook for myself. It doesn’t matter if I’m living alone, and I will eat properly just like I eat properly when I’m here with my family*’*(P8).*

### (Un)Availability of healthy and environmentally sustainable food

One-third of participants stated that there was a lack of availability of vegetarian food options or they were way more expensive than non-vegetarian options when dining out in restaurants or cafes. Therefore, some participants chose meat-based meals when dining out. However, one participant reported that she witnessed the increase in some fast-food places offering options for vegetarians and vegans due to the demand and people’s enthusiasm to purchase them. (TDF-*‘Social and Environmental influences’*).

*‘I see that a lot of people are turning vegan and vegetarian at least. They completely say no to any animal product whatsoever and that’s really nice. Because I used to work at Mad Mix. It’s like a Mexican restaurant place kind of thing. And they used to have like a lot of vegan and vegetarian options. And people really encourage and they were like, ‘Yeah, we want vegan options.’ They were not tempted by any animal products or anything*’*(P8).*

Furthermore, some participants reported eating less healthy or environmentally sustainable foods when dining out with family and friends. Participants stated that there were some options available for vegans at the markets but it was vegan junk food and reported that there was lack of local farmers markets to buy fresh fruits and vegetables. The majority of participants were eating what was available at home in the fridge or what they parents prepared. In some instances, the meals were healthy and sometimes less healthy. (TDF-*‘Social and Environmental influences’*).

*‘So sometimes when you’re out with your family or friends and having a lot of unhealthy foods because I’m a human, So definitely I feel like eating them’ (P9).*

*‘When I go out and I’m socialising with friends, it’s a bit hard to pick the healthy option’ (P16).*

A few of the participants stated that they tried or considered trying meal kit delivery services, as they believed it was easy, healthy and environmentally friendly due to reduced food miles. (TDF-*‘Behavioural regulation’*).

*‘I have considered the new meal kits that are coming out like HelloFresh, or I think there’s Marley Spoon as well, the ones which come with pre-packaged stuff. They have claims saying that meals can cost up to like less than $10 a meal, if you’ve given whatever plan or saying things like, it has a lesser carbon footprint than going to the grocery store and stuff. So I have considered trying those new meal kits and stuff, just because they seem to have good cost-effective, and environmental, and healthy benefits*’*(P18).*

### Positive and negative role modelling

Participants stated that family members, relatives and/or friends had impact on them in consuming a healthy and sustainable diet. Some participants reported that their family meals were not very healthy or environmentally friendly, but they ate it because it was prepared for them and they did not need to cook it by themselves. However, many participants described interactions with their families, relatives and friends that positively contributed to more healthy and sustainable dietary practices. For instance, a few participants explained that a vegetarian friend or family member impacted their decisions about meat intake. Family values also impacted plant-based dietary consumption. For example, some participants, or their family, practised certain religious or cultural practices that limited overall meat consumption. (TDF-*‘Social and Environmental influences’*).

*‘As occasionally, I have to follow a vegetarian diet for a few days in a year for religious purposes, because I was raised Hindu and I’m still living with my parents who practice Hinduism. So I have to be vegetarian sometimes, but mostly I eat most things*’*(P18).*

### Availability of information sources and its impact on food choices

Participants reported looking for food and nutrition-related information from a variety of sources including social media, reputable governmental sources, school and/or university courses family and friends. These sources had positive and negative impacts on their dietary behaviours. One participant reported that having a doctor in the family who encouraged consumption of a healthy diet made them more aware of what it meant to consume such a diet. (TDF-*‘Knowledge’*, *‘Social and Environmental influences’*).

*‘It’s usually my uncle because he’s a doctor and he keeps telling us that we shouldn’t eat chips. We shouldn’t unhealthy snack. He gave us all the healthy options of snacking. Sometimes he brings flavoured yogurts as a sweet dish for us after dinner, or he bring baby carrots and baby cucumbers for us, if we want to eat something. And we have a juicer in which you bring all sorts of vegetables and oranges and apples and pears and everything for healthy juices. So I would say my source of dietary information is my uncle*’*(P8).*

Public health campaigns were also influential in choosing a healthy diet.

*‘Anything that’s coming from a government source or a university source. If it’s in terms of my own research, I would usually read from a book or an article or some sort of trusted website, like usually not really blog posts. I might get ideas from it, but then I do more research on it later*’*(P5).*

Many participants believed that advertisements encouraged unhealthy food intake, and that social media fitness influencers encouraged increased consumption of meat. Also, some participants cited that they felt confident that they knew how to critically evaluate the resources due to critical thinking skills gained at university. (TDF-*‘Social and Environmental influences’*).

## Discussion

This qualitative study illuminated young Australians’ understanding of sustainable and healthy diets, current behaviours in achieving sustainable and healthy diets and barriers and opportunities associated with adopting or maintaining sustainable and healthy dietary behaviours. The findings contribute to the current literature on consumer behaviour regarding sustainable diets in several ways.

First, they suggest that some progress has been made in the last few years in terms of increased consumer understanding and awareness of the impact of foods on the environment and health. Most of our participants were familiar with the concept of healthy and sustainability diets, and often cited production and overconsumption of animal-derived foods, mainly referring to meat intake, as having a negative impact on the environment and health. This contrasts with previous studies conducted in Australia^(15–17)^, New Zealand^(27)^ and other Western countries^(28,29)^ that described limited awareness. A systematic literature review conducted in 2017 on sustainable protein intake found that consumer awareness of the impact of meat production and consumption on the environment and motivation to reduce it were low^(28)^. It is important to note that the understanding of sustainable and healthy diets among our participants was mixed – some had not heard of the concept and others had an understanding that was not necessarily comprehensive; our participants principally focused on health aspects, reduction of animal-derived foods (mainly meat) and food waste and packaging. This aligns with other studies that found participants focus more on food choices impacting their health rather than environment except for food waste^(15,16,30,31)^. Therefore, there is still a need to educate the public on what constitutes a sustainable and healthy diet, how it looks on the plate and educate on ecolabelling^(32)^ to help young adults (and others) make informed food choices.

Second, our participants demonstrated increased motivation to change to more sustainable and healthy dietary behaviours in comparison with most previous studies^(15,28)^. This aligns with Kemper’s study stating that young New Zealanders were motivated to reduce meat intake for environmental and health reasons^(27)^. Our participants cited numerous strategies to reduce intake of certain foods with high environmental impact – eliminating consumption, making alternative food choices (e.g. poultry instead of red meat) and modifying their diets to maintain ‘balance’ over time. However, although many of our participants had motivation, they also indicated several individual and environmental barriers impacting behaviour change such as low food literacy, including cooking skills, lack of time, lack of information on plant-based recipes, lack of plant-based options when dining out and costs associated with plant-based diets. These barriers align with what has been reported in previous studies conducted in Western countries^(28,33–35)^.

Limited food preparation and cooking skills impacted our participants’ meal preparation, food choices and purchasing behaviours. Food literacy, including adequate food preparation and cooking skills, has been associated with healthier dietary behaviours and informed food choices^(36–38)^. However, declining food preparation and cooking skills among younger generations has been noted in the literature^(18,37,39–41)^. Although an increase in health promotion interventions promoting reduction in meat intake has shown reduced intentions to consume meat, there is limited evidence showing whether actual behaviour change was made or sustained^(42)^. If individuals do not have the skills or abilities to act on their intentions, this could be a major limitation to improving diets. Our participants expressed some difficulties in preparing vegetarian dishes which aligned with findings from another study^(33)^. Therefore, there is a need for public education campaigns and social marketing interventions to include opportunities for young adults to increase their food literacy, including their food preparation and cooking skills. In addition, there is a need to develop recipes which promote sustainable and healthy dishes and ingredients that are cost- and time effective. Future research is needed to evaluate these different individually focused behavioural interventions to determine their efficacy in terms of changing intentions, food literacy and food choice, improving skills and abilities, and ultimately whether they contribute to the consumption of sustainable and healthy diets among young adults.

Availability and accessibility of plant-based food options and costs of fresh and local produce including fruits and vegetables also impacted our participants’ food choices. Although educational awareness-based campaigns can increase knowledge and motivation on sustainable practices, on its own it is thought to be insufficient to change behaviour^(43)^. It has been suggested that behaviour change can be achieved by altering environments within which people make choices^(44)^. There has been increase in choice architecture and nudging interventions such as menu manipulation and restructure of food environments to motivate people to make pro-environmental food choices^(45,46)^. A recent systematic literature review synthesised the evidence of interventions aiming to reduce the demand for meat and found that most promising interventions were those which reduced portion sizes of meat servings, provided meat alternatives with supporting educational material or manipulated the sensory properties of meat or meat alternatives. Additionally, there was evidence of some effectiveness of interventions that repositioned meat products to be less prominent at the point of purchase^(45)^. Our findings show that young Australians might be receptive to these strategies, as some of our participants already implemented these strategies in their own diets, and many described being open to or interested in affordable alternatives at food retailers they frequented. This suggests that there are opportunities for food and hospitality industries, including restaurants, cafes and take-away outlets, to engage in sustainable food practices, for example by offering more plant-based options at affordable prices. This also suggests possible opportunities for farmers and community-based initiatives, such as fruit and vegetable cooperatives^(47)^, to offer fruits and vegetables at competitive prices in order to increase selection of more plant-based foods. Choice architecture, nudging strategies and community-based interventions can be promising approaches to create enabling food environments and for changing dietary behaviour towards more sustainable and healthy diets. However, there is a need to test different strategies and changes to food environments to determine their contribution to increased adoption of sustainable and healthy diets.

It is important to acknowledge both the strengths and limitations of the current study which may have had an impact on our findings. We used the TDF^(23)^ for data analysis, which provided a strong theoretical basis for this research and allowed us to build a nuanced understanding of behaviours impacting on the consumption of sustainable and healthy diets. This research included participants at different behaviour change stages, some of whom were aware and practising some aspects of a sustainable and healthy diet, and others who were either not aware or not practicing it. This allowed the exploration of barriers and enablers at different stages of behaviour change. A number of TDF constructs should be taken into account in order to develop successful behaviour change interventions aiming to promote sustainable and healthy diets among young adults. Future interventions should help participants to increase their *‘knowledge’* on sustainable diets, *‘beliefs about consequences’* in adopting sustainable diets, *‘behaviour regulation’* and *‘social professional role and identity’*; develop food preparation and cooking *‘skills’* and provide supportive environment- *‘social and environmental influences’*. We recruited young adults residing in Australia, therefore the generalisability may be limited. However, our findings aligned with those from many other similar settings. We also used the online Zoom platform to interview participants which enabled us to recruit participants from different geographical locations across Australia who were affiliated with the university. Young people have demonstrated an increased interest in environmental issues^(48)^, therefore the experiences, barriers and enablers in achieving a sustainable and healthy diet might be similar. Furthermore, selection bias may exist given that our participants chose to participate in this research; they may have more interest and knowledge in sustainable and healthy diets. We tried to minimise this potential bias by designing the recruitment materials stating that we were looking for participants to share their experiences on their dietary behaviours. Finally, our participants were highly educated individuals (either having an undergraduate University degree or being currently enrolled students), and the majority of our participants were females. Therefore, this may not reflect the views of general young Australian population.

## Conclusion

The present study showed that young Australians are aware of some aspects of a sustainable and healthy diet. Our findings also suggest that young Australians are motivated to adopt more sustainable and healthy dietary practices, but might not be able to do so unless several individual and environmental barriers are addressed. Young adults could benefit from experiential behavioural change interventions that aim to increase their food literacy around sustainable and healthy diets. These include food preparation and cooking skills that would enable preparation of sustainable and healthy meals, as well as supportive food environments that help facilitate informed and environmentally friendly food choices. Our findings inform future research for development and evaluation of both individual and micro-environmental-based interventions to encourage greater adoption of sustainable and healthy diets among youth and other Australian populations.

## Supplementary material

For supplementary material accompanying this paper visit https://doi.org/10.1017/S1368980022001513. click here to view supplementary material
