# Supplementary material for: Exploring young Australians’ understanding of sustainable and healthy diets: a qualitative study
Source: Public Health Nutr. 2022 Jul 7;25(10):2957–69. doi: 10.1017/S1368980022001513 (PMC9991849; doi:10.1017/S1368980022001513)
Supplement: Supplementary file 1 [file S1368980022001513sup.zip › S1368980022001513sup001.docx]

**Supplementary file 2****:** Theoretical Domains Framework (TDF) and selected illustrative quotes

| **TDF construct and the number of participants mentioned it** | **Selective quotes** |
| --- | --- |
| 1-**Knowledge**  (An awareness of existing of something) N=22 | *"I am actively trying to cook with less meat and buy less meat because I know that industry produces a lot of greenhouse gases"(P1).*  *"Sustainable diet would mean not just what you're eating, but also where it's produced, how much packaging it has"(P5).*  *"for me personally, a sustainable, healthy diet is one where I can reduce my food waste, reduce my plastic consumption, eat a wide variety of whole foods, as well as incorporate some discretionary items and be satisfied at the end of the day"(P12).*  *"Environmentally sustainable diets would be, not maybe getting takeout containers every day of the week, or buying things in bulk. So, you're not going to the grocery store every day of the week. So not using your car, not making seven trips every week, that sort of thing. So, thinking of environmentally sustainable practices, how to incorporate them into your eating as well. Hope that makes sense."(P18).*  *"So, I specifically know that beef, like cattle is very heavy on the environment in terms of CO2 emissions, I think it is. And a lot of the growing of crops in raising of cattle requires a lot of water. I know that's the things that come to mind. And in terms of the environment, I think it's a lot to do with the process as well. If you're growing a crop, how the land is treated and the actual growing process and harvesting and things like that. I don't really know much at all, to be honest"(P20).* |
| 2-**Skills**  (An ability or proficiency acquired through practice) N=12 | *"When I became a vegetarian, so that changed pretty rapidly. I wasn't a great cook back then, so I probably just ate a lot of carbs and not a lot of vegetables, and wasn't particularly healthy. So, I gradually learned how to cook, and cooked better food for myself"(P6).*  *"I prepare most of my, try preparing as much as I can by myself and whatever I've sent you I have prepared myself"(P10).*  *"When I cook, usually I cook for two days at one time, especially if I'm really busy. So, if I cook for two days at one time, it helps me not to eat unhealthy like noodles and stuff. That really helps, I guess"(P15).*  *"If I prepare it from home, like I'm going to work. If I prepare food that obviously makes me eat healthier. Cause I don't buy food when I'm out, so it's all from home"(P19).*  *"I'll cook mostly for myself if I don't want to eat what everyone else is eating"(P20).* |
| 3-**Social professional** **role and identity**  (A coherent set of behaviours and displayed personal qualities of an individual in a social or work setting) N=13 | *"I've got an Asian background and I guess brown rice and things like that are things that are healthier"(P2).*  *"It has to be something which is true to your roots, I mean, from whichever ethnic background you're from. If that's what you're used to eating from your childhood, you cannot just completely change. If you have been a non-vegetarian your whole life, you cannot turn into a vegan overnight. A sustainable diet would be something which comes from your roots"(P4).*  *"My first preference is chicken. My second preference is ham and I ignore beef because of the religious reasons that Indians don't eat beef. But if something comes really good in a beef patty, then I won't say no to it. But I would obviously look for other options and avoid beef as long as I can"(P8).*  *"I'm a Bangladeshi, We Bangladeshi's are usually very big fans of fish. I don't like fish myself to be honest, my culture likes a lot of fish. I don't like fish at all. The only kind of fish that I eat are sea fish"(P10).*  *"Probably just the habit, the habit of in Mexico, there weren't many options. It was all a lot of meat, and telling people about it, and yeah, that was more of a challenge, like people accepting that you're not going to eat whatever"(P13).* |
| 4- **Beliefs about** **capabilities**  (Acceptance of the truth, reality or validity about an ability, talent or facility that a person can put to constructive use) N=0 |  |
| 5-**Optimism**  (The confidence that things will happen or the best or that desired goals will be attained) N=4 | *"I just take everything that I read with a grain of salt. I just take information that I've heard multiple times more seriously, than things that I've heard once or twice"(P6).*  *"I don't know. I just trust it [information source]. I've never thought it like that, that why should I trust it? I just do. Real plant products, they just have a why that you should trust them. I don't know. I just do"(P8). "The information that I get from Google, I trust most of it because I look at the website itself, I think in other websites where they're not, they don't give you false information"(P10).*  *"not everything that they state is a 100% correct. Same goes for our grocery stores not every product label is a 100% accurate. There's a lot of loopholes and people make use of it. So, it's important to keep an eye out on the news, that's what I believe"(P10).*  *"I guess, applying some criticality to it, seeing who is saying it, why would they be saying it, and if it aligns with other things I've heard. So if the government came out tomorrow saying, smoking is great or maybe not smoking, but like sugar is really good for you, to have as much sugar as possible, I'd be like, "Well, what's happening here?" (P18).* |
| 6- **Beliefs about** **consequences** (Acceptance of the truth, reality or validity about outcomes of a behaviour in a given situation) N=16 | “*I don't think the environmental issues should be pushed to the individual. I think it's the corporations, it's the entire meat industry that should be held accountable for environmental change. I know that my food choices support that regime, but if I were to change it, then I don't believe that the whole industry would change"(P1).*  *"It's not just for the physical aspect but more the mental aspect, because the better you eat, the higher in nutrients you are, which means that you're better able to process information and remember, and it regulates mood"(P5).*  *"Knowing that in the future my health is going to be pretty dependent on how I choose to live my life now. I know that the older you get, the more difficult it is to maintain a healthy lifestyle if you haven't started younger"(P6).*  *"I try avoiding beef, even though I eat beef all the time, but I do eat beef a little less because I know that beef cultivation is still harmful towards our environment and I'm very against animal abuse"(P10).*  *"I think eating healthy would like it makes your life like your lifestyle much more productive, having junk food makes you lazy, because I've experienced from eating, eating junk I end up not doing anything any work done"(P22).* |
| 7-**Reinforcement**  (Increasing the probability of a response by arranging a dependent relationship or contingency, between the response and a given stimulus) N=1 | *"Knowing that in the future my health is going to be pretty dependent on how I choose to live my life now. I know that the older you get, the more difficult it is to maintain a healthy lifestyle if you haven't started younger"(P6).* |
| 8-**Intentions**  (A continues decision to perform a behaviour or a resolve to act in a certain way) N=8 | *"Not so much eggs, because I usually go through cycles of either I want to eat them or I don't"(P6).*  *"But I would like it to maintain, I guess, the environment, the qualities of our unique Australian environment to be maintained as much as I can, or much as I wish. And I do like to minimize the impact of what I eat, to allow our environment to flourish for the feature, for not only myself and the current generation, but also for future generation to appreciate the biodiversity and the environmental diversity that we have in Australia, and not just Australia but worldwide too"(P7).*  *"Environmental effect is something that I personally take very seriously. It has altered my diet quite a bit. I preach altering your diet for environmental issues. So I also run, I have a foundation back home in Bangladesh where we encourage children to be more sustainable in their movements. We educate them about sustainability as well"(P10).*  *“I think, I need to take care of my body, it's the only one I have, as well as taking care of it internally for my immunity and general healthiness, as well as how I look outside appearance-wise. I don't want to have a beer belly by the time I'm 30. I want to try and stay as healthy looking as possible"(P18).*  *"But now I tend to be more mindful about like how my body works, and I only eat when I'm truly hungry basically just listening to my body more"(P21).* |
| 9-**Goals**  (Mental representations of outcomes or end states that an individual wants to achieve) N=7 | *"I do a meal plan at the start of the week or just before I go shopping, and that helps me save a bit of money"(P1).*  *“My weight. I know that the BMI range that I'm in, it's not in the normal side, it's slightly overweight, so I am trying to cut down my food or trying to incorporate more healthier choices"(P4).*  *"I'm pretty sure when I moved back to Sydney, when this COVID thing settles, and I have a proper job in Sydney, because I don't have proper work in Sydney. I work in Hong Kong. So if I get a proper job in Sydney, I have made a promise to myself that I'll cook for myself. It doesn't matter if I'm living alone, and I will eat properly just like I eat properly when I'm here with my family"(P8).*  *"It's very important to be mindful of the macronutrients. I count my calories daily; I try to stay in a calorie deficit if I can. That's what I'm trying to do at the moment, because I'm cutting my weight"(P10).*  *"Number one, it's also appearance. If I'm going to be very honest with you, it's about appearance. Secondly, it's about keeping our system clean because I noticed a big change in my body because I was a smoker, to be honest. And before I started working out, when I wasn't doing my, when I was doing my cardio. So when I was walking on the treadmill or running on the treadmill, it took a big toll on my body. I was also eating very, not clean. So I was eating very dirty. I was eating out of carbs I was eating a lot of bad protein. I was eating fatty foods and had a big effect on my body which I personally realized once I started working out every day. And that's why I decided to make the switch and I encourage everyone to make the same switch"(P10).* |
| 10- **Memory, attention and decision processes**  (The ability to retain information, focus selectivity on aspects of the environment and choose between two or more alternatives) N=2 | *"There are all these foods that I really liked when I was a child, that I wasn't allowed to eat when I was a child, as well. So, it can be really, really tempting to just be like, "Oh, I'm just going to treat myself." But then if it becomes every week thing, then it's not treating yourself. That's just part of what you're eating"(P6).*  *"When I was in year 10, I was really fat and I didn't really care about what I was eating. I used to go to our school canteen and eat all sorts of junk foods and I gained a lot of weight. But in year 11, I really stretched myself out. I did a lot of workout and I eat healthy and I lost about 10 KGs of weight"(P8).* |
| 11- **Environmental context and resources**  (Any circumstance of a person's situation or environment that discourages or encourages the development of skills and abilities, independence, social competence and adaptive behaviour and person's situation impact on food or environmental situation impact on food) N=22 | *"Going out to eat, sometimes meat options are the majority of the options that are available. So when I go out to eat, for example, meat probably more of a popular option than when I am at home"(P2).*  *"A lot of my friends are really busy; they all work more hours than I do. And so, the idea of them cooking when they get home, it's really difficult. Whereas, because I do most of my work from home, I have more time to cook"(P6).*  *"I used to love cakes a lot and yeah, and muffins and all that whip cream. So sometimes you just feel tempted to just going back to all that and it's easy to get it. It's easy, it's even affordably cheaper to buy it as compared to when you want to do a high protein diet, where you have to get your meat, you have to put in your vegetables, you have to put in your carbohydrate. You can just have a cake and eat, that's very easy if you ask me. It's so different from having to cook everything else to make a proper dinner or a problem now with all the nutrients"(P14).*  *"It's hard for me to stick to a diet. Unfortunately, since the effects aren't immediate and they aren't incredibly noticeable all the time, it's hard for me to stick to a diet and keep eating in a way that I should be, or I feel like I should be eating"(P18).*  *"I tried to prepare as much as possible (food) because It's quite expensive outside but I make, make sure once a week, I go out and I like I eat outside"(P22).* |
| 12-**Social influences**  (Those interpersonal processes that can cause individual to change their thoughts, feelings or behaviours) N=21 | *"When I was obviously much younger, my mom cooked everything for me"(P6). "As I got older, as I didn't cook at the time, my mom was the chef. As she got more easy with work, the selection of food has been the variety, I guess, slimmed down considerably to something that's more quick and convenient"(P7).*  *"Most recently me and my sister have been watching documentaries on how the meat industry works. And I guess the nature of how they're doing it isn't really good for the environment and stuff like that. We're trying to be more sustainable in where we eat. So that's, I guess the reason why we've shifted towards more vegetarian options, but at the same time, we also know that there's probably better health benefits from that as well "(P2).*  *"Think it's pretty similar, especially between my sister and mum. Probably the only person that's a bit different would be dad. He's not really concerned about healthy eating. But I think amongst us girls, it's probably quite similar"(P5).*  *"My uncle, one of them is a doctor, so he really motivates us to eat healthy food and do healthy snacking. So our snacks usually at home is salad. We cut some carrots and cut some tomatoes and put some salt and pepper in it and that's our healthy snacking"(P8).* |
| 13- **Emotion**  (A complex reaction pattern, involving experiential, behavioural, and physiological elements, by which the individual attempts to deal with a personally significant matter or event) N=19 | *"My mental health definitely plays a role in it. There are days or weeks where I'm just not motivated to do much. And it's hard to think to yourself, well, I'm going to prepare a healthy meal"(P1).*  *"I feel really bad mentally if I eat like chicken or pork or beef, maybe like two to three consecutive days. It happens only if I'm out. So if I'm with my family living, we have the home cooked meal, which is always vegetarian. But, if I'm visiting uni and I'm meeting people, so I might consume something which has non-veg in it, so I would feel really bad. And maybe psychologically for the environment and also for my body that I've consumed a lot of meat. So what I would usually do that I won't eat meat for next month"(P8).*  *"Eating healthy brings me joy and I feel good about eating healthy and it's something that relates to my health as well"(P9).*  *"I think it's very linked for me to just feeling good, because, for example, when I was eating all the food bank stuff, because it's not fresh, I was feeling more lethargic and more heavy. So, first thing is it makes me feel good and that it's fresher"(P13).*  *"So I do feel better. It's part of the mind as well, you feel guilty when you eat junk food, but you do notice it in your energy levels. I don't feel as much bloated. Say if I eat a pizza, I would feel bloated after that, but after eating a healthy meal, I don't feel as bloated. And say if I had a pizza, I wouldn't feel hungry for a very long time, and it disrupts my cycle"(P15).* |
| 14- **Behavioural regulation**  (Anything aimed at managing or measured actions) N=22 | *"I never go shopping hungry because I find that I want instant satisfaction and if I go on full stomach, then I'm not going to pick up the pre-packaged or processed bullshit"(P1).*  *"I refuse to buy wine that's been imported from overseas just because of the carbon footprint that it has to get it into Australia, when we have really good wine here in Australia"(P3).*  *"I was told that acne is directly related to the proportion of dairy that I consume, which is why I'm trying to cut down dairy. But cutting that down has been a huge problem for now, because I have to restrict not just milk, but milk products as well, so that is, cheese, yogurt and cream cheese, or bakery products or anything that even has a certain amount of dairy I have to cut down on. That has been a problem because I like my coffee a lot"(P4).*  *"I sometimes try to be careful about how much additional fat I add to things. If I'm cooking with butter or oil, to just be careful about how much I'm using. Especially when I'm frying, doing a stir-fry with vegetables and stuff, if I feel I'm using too much oil, to maybe just put in a little bit of water, instead of adding more oil"(P6).*  *"Restricting my carbohydrate intake. So this is one of the thing that I'm still struggling because I do love carbs, but because my physical activity is not as much as I used to have since young"(P9).* |
