# Supplementary material for: Exploring young Australians’ understanding of sustainable and healthy diets: a qualitative study
Source: Public Health Nutr. 2022 Jul 7;25(10):2957–69. doi: 10.1017/S1368980022001513 (PMC9991849; doi:10.1017/S1368980022001513)
Supplement: Supplementary file 1 [file S1368980022001513sup.zip › S1368980022001513sup002.docx]

**Supplementary file 1**

**Demographic and introduction type of questions:**

- What is your age, sex, race, marital and employment status?
- What is the highest level of education you have completed?
- What is your occupation?
- How many people live in your household? How many children live in your household?
- Do you have any dietary restrictions or follow any diet? (E.g. vegetarian, paleo diet, Mediterranean diet etc. or reasons such as health condition, pregnancy, religious beliefs etc.).
- In the last few weeks, did you experience any financial barriers in regards to purchasing food? (E.g. running out of food and being unable to purchase more due to cost; having difficulty ensuring a constant food intake is sustained).

**Sustainable and healthy diet related questions:**

Thanks for sending the pictures of your dinners. Did you prepare all of this by yourself or did you purchase it? How did you decide what to eat/cook for each of this meal? What factors had an impact on your decisions regarding dinner choices? Where did you get the ingredients from, why from these sources? Do you usually prepare these types of meals?

In your own words, how would you describe a sustainable and healthy diet? Could you describe how you think a sustainable and healthy (dinner) plate looks like?

Probe: If you had a standard dinner plate and were serving yourself, what would a healthy and sustainable diet look like on the plate (how would you portion the plate)? Tell me more about why that is.

Probe: Do you consume snacks? What about healthy and sustainable snacks?

What does it mean to you to eat healthy?

Probe: Are there certain foods you eat more or less of for health reasons? Tell me more about that. What about drinks?

Probe: What motivates you to eat or drink healthy?

Probe: What strategies do you use to help you to eat or drink healthy? OR what helps you to eat healthy?

Probe: Do you face any challenges when trying to eat healthy? OR Do you have any difficulties when trying to eat healthy?

Probe: How does your view about healthy eating compare to the views of other people you know?

- Have you made any changes to the way you eat? If yes, then, how and why did your eating change?
  - Probe: How is what you eat now different from what you used to eat when you were growing up? Why is that? How do you feel about those changes? Did you have any difficulties in changing your diet? What were they?
- Have you ever heard about the impact of different foods on the environment? If yes, then, what have you heard?
  - Probe: If YES, then has it or would it change the foods that you eat or the frequency in which you eat some foods? How so? Why or why not?
  - Probe: If NO, then if you heard that certain foods had a negative impact on the environment, would you change what you ate? Why or why not?

How does a healthy diet align with your views on a sustainable diet, that you just described?

Probe: Are there certain foods you eat more of or less for environmental reasons? Tell me more about that.

Probe: What do you think about how your food choices contribute to the environment? For example, looking at your dinner pictures, do you think any of your food choices contribute to the environment, how?

Probe: What motivates you to eat foods with low environmental impact?

Probe: If striving for sustainable diet, then, what strategies do you use to help you to eat more environmentally sustainable foods OR what helps you to eat foods with low environmental impact?

Probe: If striving for sustainable diet, then, do you have any difficulties, or do you face any challenges when you are trying to eat foods that have low environmental impact?

- What role do meat, fish, dairy, or eggs play in your diet?
  - Probe: Have you ever tried to change your meat intake? Have you ever tried any foods that are intended to be meat substitutes? If yes, then, what were they? What do you think about them? Would you eat them again? Why or why not?
  - Probe: If eats meat, then, are there any kinds of meat you eat more than others? Why is that? How many days per week do you eat red meat (e.g. beef or lamb)?
  - Probe: If tried to reduce foods listed above, then Did you face any difficulties in reducing [red meat, dairy etc.] consumption?
- Where do you get information about what foods you should eat? (E.g. other people, media, news, friends, doctors, nutritionists)
  - Probe: What do you think about that information? How do you feel about the information you learn from [participant listed] sources?
  - Probe: How do you know what information to trust? How do you determine when information is reliable? Are there sources you avoid because you think they are untrustworthy? What are those sources?
- Is there anything else you would like to add?
